# Effects of Long Term Antibiotic Therapy on Human Oral and Fecal Viromes

**DOI:** 10.1371/journal.pone.0134941

**Published:** 2015-08-26

**Authors:** Shira R. Abeles, Melissa Ly, Tasha M. Santiago-Rodriguez, David T. Pride

**Affiliations:** 1 Department of Medicine, University of California, San Diego, La Jolla, CA, 92093, United States of America; 2 Department of Pathology, University of California, San Diego, La Jolla, CA, 92093, United States of America; Charité, Campus Benjamin Franklin, GERMANY

## Abstract

Viruses are integral members of the human microbiome. Many of the viruses comprising the human virome have been identified as bacteriophage, and little is known about how they respond to perturbations within the human ecosystem. The intimate association of phage with their cellular hosts suggests their communities may change in response to shifts in bacterial community membership. Alterations to human bacterial biota can result in human disease including a reduction in the host's resilience to pathogens. Here we report the ecology of oral and fecal viral communities and their responses to long-term antibiotic therapy in a cohort of human subjects. We found significant differences between the viral communities of each body site with a more heterogeneous fecal virus community compared with viruses in saliva. We measured the relative diversity of viruses, and found that the oral viromes were significantly more diverse than fecal viromes. There were characteristic changes in the membership of oral and fecal bacterial communities in response to antibiotics, but changes in fecal viral communities were less distinguishing. In the oral cavity, an abundance of papillomaviruses found in subjects on antibiotics suggests an association between antibiotics and papillomavirus production. Despite the abundance of papillomaviruses identified, in neither the oral nor the fecal viromes did antibiotic therapy have any significant impact upon overall viral diversity. There was, however, an apparent expansion of the reservoir of genes putatively involved in resistance to numerous classes of antibiotics in fecal viromes that was not paralleled in oral viromes. The emergence of antibiotic resistance in fecal viromes in response to long-term antibiotic therapy in humans suggests that viruses play an important role in the resilience of human microbial communities to antibiotic disturbances.

## Introduction

The human microbiome is a highly complex community of microorganisms consisting not only of diverse bacteria, archaea, and eukaryota (fungi), but also of an immense population of viruses [1,2,3,4,5,6]. The viral communities existing within certain body sites, such as within the oral cavity and within the colon, appear to consist largely of bacteriophage, although eukaryote viruses have also been identified as members of these communities [1,5,7]. Viruses, including bacteriophage, may play an important role in human mucosal health [8] and immunity [9]. Viruses are important factors in the ecology of local microbial ecosystems and can have various effects on microbiota, such as impacting microbial diversity in a community [10], stimulating evolutionary change in bacterial hosts [11,12,13], and possibly providing selective advantages to bacterial hosts [14,15,16].

There are several factors limiting the study of viral communities through metagenomics. Current methods of viral isolation in preparation for sequencing often exclude certain viruses from virome sequencing, such as RNA viruses or large viruses. The use of multiple displacement amplification (MDA) when quantities of viral DNA recovered are limited also can introduce biases into viromes [17,18]. Another potentially more significant limitation in the analysis of viral communities has been a relative lack of tools available for characterizing their ecology and their diversity compared to the analysis of bacterial communities which can be done with a number of tools such as QIIME [19] and Mothur [20], which have greatly facilitated the characterization of bacterial biota using 16S rRNA. There is a significant need to implement analogous tools for the analysis of viral metadata to define the membership of complex microbial ecosystems and their interactions with local environments. Current widely available tools for viral analysis include MetaVir, a web-based tool for the annotation of viral metagenomes [21]. MetaVir can provide estimates of gene richness in viromes by clustering genes based on their genetic diversity. However, ecological estimates are based on gene sequences rather than individual viruses. Another tool, PHACCS, estimates viral diversity based on predictions of population diversity using contig spectra, but requires estimates of mean virus genome lengths in the population to predict diversity [22].

While antibiotics do not target bacteriophage directly, they target their bacterial hosts. Thus human viral ecology might be expected to reflect changes in bacterial ecology, though relative abundances of bacteria on human body surfaces do not necessarily predict the relative abundances of their viruses [1]. In murine models, certain antibiotics have been shown to increase the reservoir of antibiotic resistance in fecal viral communities [23]. Antibiotics may also result in the induction of prophage as has been demonstrated to occur in the swine gut [24,25]. The induction of prophage has implications for the transmission of antibiotic resistance genes to other acceptor strains, which was demonstrated in these same studies. The production of toxins from prophage [26] also has significant implications for human health and disease, as it has been shown that Shiga Toxin Producing *Escherichia coli* respond to antibiotics by increasing their production of Shiga-toxin-1 (Stx1) [27,28], which is known to be involved in the development of dysentery [29] and hemolytic uremic syndrome [30] in humans.

Relatively little is known about the ecology of human viromes and how viral communities may respond to ecological perturbations such as caused by antibiotic exposure. We hypothesized that viral communities in the human body would be sensitive responders to the powerful selective pressures imposed by antibiotics, potentially as a reflection of changes in bacterial biota. We recruited a cohort of subjects taking a 6-week course of antibiotics and sampled their saliva and feces longitudinally to examine the effects of long-term antibiotics on human viral ecology. Our goals were to: 1) discern whether there are significant differences in human viral communities on these distinct body surfaces in the same subjects, 2) utilize techniques to characterize viral diversity to examine the effects of long-term antibiotics on human oral and gut viral communities, and 3) discern whether the use of antibiotics in humans results in an increase in the reservoir of antibiotic resistance in viral communities in the gut and oral cavity.

## Materials and Methods

### Human Subjects

This study was approved by the Institutional Review Board of the University of California, San Diego. Each subject signed informed consent indicating his or her willingness to participate in this study. Subjects donated saliva and fecal samples on day 3, week 2, and week 6 of intravenous antibiotic therapy (S1 Table). Only 1 of the 4 subjects remained hospitalized during their antibiotic therapy, while the other 3 subjects received their antibiotics at home. This subject remained hospitalized due to difficulty in arranging home antibiotics and not due to illness severity. All subjects were able to consume normal diets. A separate group of subjects donated saliva and fecal samples over the same time period, however, this group received no antibiotic intervention or placebo. Fecal samples were collected when the subjects were able to produce them, and saliva samples were produced at the same time to reduce the time period between collecting each sample type. Samples consisted of a minimum of 3mL of unstimulated saliva and 1 gram of feces. Each was frozen immediately at -20°C prior to use in this study. The fecal sample at 3 days for subject #2 was not processed in this study because it was improperly preserved. Exclusion criteria for the study included any antibiotic treatment in the 6 months prior to enrollment.

### Preparation and sequencing of metagenomic libraries

Fecal viromes were prepared by diluting 0.4g of feces in 4ml of SM buffer. The fecal samples were vortexed vigorously for 40 minutes to separate viral particles, and the supernatant was treated in an identical manner to that of saliva. Fecal and saliva samples were centrifuged at 4,000 x g for 10 minutes to pellet the remaining solid material, sequentially filtered using 0.45μm and 0.2μm filters (VWR, Radnor, PA) to remove cellular and other debris, and then purified on a cesium chloride gradient according to previously described protocols [1]. Only the fraction with a density corresponding to most known bacteriophage [31] was retained, further purified on Amicon YM-100 protein purification columns (Millipore, Inc., Bellerica, MA), treated with 2U of DNase I (Roche Diagnostics, Indianapolis, IN), and subjected to DNA purification using the Qiagen UltraSens Virus Kit (Qiagen, Valencia, CA). Resulting DNA was amplified for 16 hours using GenomiPhi HY MDA amplification (GE Healthcare, Pittsburgh, PA), fragmented to roughly 200 to 400bp using a Bioruptor (Diagenode, Denville, NJ), and utilized as input to create libraries using the Ion Plus Fragment Library Kit according to manufacturer’s instructions. Libraries then were sequenced using 314 or 316 chips on an Ion Torrent Personal Genome Machine (PGM; Life Technologies, Grand Island, NY) [32] producing an average read length of approximately 215bp for each sample.

### Analysis of viromes

We trimmed each sequence read according to modified quality scores of 0.5 using CLC Genomics Workbench 4.65 (CLC bio USA, Cambridge, MA), removed any low complexity reads that had a stretch of ≥10 consecutive homopolymers, and removed any reads with substantial length variation (<50 nucleotides or >300 nucleotides) or ambiguous characters prior to further analysis. Each virome was screened for contaminating bacterial and human nucleic acids using BLASTN analysis (E-value <10^−5^) against the Ribosomal Database Project 16S rRNA database [33], and the human reference database (ftp://ftp.ncbi.nlm.nih.gov/genomes/H_sapiens/). Reads homologous to human sequences were removed prior to further analysis. Remaining reads were assembled using CLC Genomics Workbench 4.65 based on 98% identity with a minimum of 50% read overlap; a highly stringent set of criteria developed to discriminate between highly related viruses [34]. Because the shortest reads used were 50 nucleotides, the minimum tolerable overlap was 25 nucleotides, and the average overlap was no less than 107 nucleotides depending on the characteristics of each virome. The consensus sequence for each contig was constructed according to majority rule, where for any nucleotide position where polymorphisms existed, the nucleotide that represented ≥50% of the nucleotides at that position amongst the sequence reads was used to build the consensus sequence. Any contigs <200 nucleotides or with ambiguous characters were removed prior to further analysis.

Contigs were annotated using BLASTX against the NCBI Non-redundant (NR) database with an E-value cutoff value of 10^−5^. Specific viral homologues were determined by parsing BLASTX results for known viral genes including replication, structural, transposition, restriction/modification, hypothetical, and other genes previously found in viruses for which the E-value was at least 10^−5^. Each virome contig was individually annotated using this technique; however, if the best hit for any portion of the contig was to a gene with no known function, lower level hits were used as long as they had known function and still met the E-value cutoff. The annotation data were compiled for each subject and used to determine the relative proportions of assembled contigs that contained viral homologues. The profiles of the putative hosts for the phage based on phylum level BLASTX best hits were created for each donor and sample type. We utilized the number of reads used in the assemblies of each contig to determine the relative abundance profiles of different phyla to compensate for viruses that may be more abundant than others. This technique prevented reads involved in the assembly of the same virus contigs from being assigned to different putative host phyla based on different BLASTX homologies. Determination of the relative abundances of virus families were determined by BLASTX analysis of the SEED database using MG-RAST [35].

Analysis of shared homologues present in each virome was performed by creating custom BLAST databases for each virome, comparing each database with all other viromes using BLASTN analysis (E-value <10^−10^). Principal coordinates analysis (PCOA) was performed on homologous virome contigs with binary Sorensen distances using QIIME [19]. Determination of the proportions of viral contigs putatively involved in antibiotic resistance was performed using BLASTX analysis (E-value <10^−30^) against the Comprehensive Antibiotic Resistance Database (CARD) [36]. We eliminated any homologous reads that could result in antibiotic resistance through mutation, as the presence of homology among those reads may not result in antibiotic resistance. These included DNA topoisomerases, DNA gyrases, DNA polymerases, RNA polymerases, ribosomal RNA, and ribosomal proteins, which resulted in a significant reduction in the proportions of homologous proteins involved in resistance to quinolone and rifamycin antibiotics. Homologues were classified according to antibiotic classes beta lactamases, penicillin binding proteins, macrolides, tetracyclines, quinolones, sulfonamides, aminiglycosides, glycopeptides (vancomycin), chloramphenicol, fosfomycin, and multi-drug efflux pumps capable of transporting multiple antibiotic classes. All homologues were compiled by proportion of total virome contigs per subject and relative proportions compared among all subjects by antibiotic use status by t-test using Microsoft Excel 2007 (Microsoft Corp., Redman, WA).

### Analysis of 16S rRNA

Genomic DNA was prepared from the feces of each subject and time point using the Qiagen QIAamp DNA Stool Mini Kit (Qiagen, Valencia, CA). We amplified the bacterial 16S rRNA V1-V2 hypervariable region using the forward primer 8F (AGAGTTTGATCCTGGCTCAG) fused with the Ion Torrent Adaptor A sequence and one of 23 unique 10 base pair barcodes, and reverse primer 357R (CTGCTGCCTYCCGTA) fused with the Ion Torrent Adaptor P1 from each donor and sample type [37]. PCR reactions were performed using Platinum High Fidelity PCR SuperMix (Invitrogen, Carlsbad, CA) with the following cycling parameters: 94°C for 10 minutes, followed by 30 cycles of 94°C for 30 seconds, 53°C for 30 seconds, 72°C for 30 seconds, and a final elongation step of 72°C for 10 minutes. Resulting amplicons were purified on a 2% agarose gel stained with SYBR Safe (Invitrogen, Carlsbad, CA) using the MinElute PCR Purification Kit (Qiagen, Valencia, CA). Amplicons were further purified with Ampure XP beads (Beckman-Coulter, Brea, CA), and molar equivalents were determined for each sample using a Bioanalyzer 2100 High Sensitivity DNA Kit (Agilent Technologies, Santa Clara, CA). Samples were pooled into equimolar proportions and sequenced on 314 chips using an Ion Torrent PGM according to manufacturer’s instructions (Life Technologies, Grand Island, NY) [32]. Resulting sequence reads were removed from the analysis if they were <180 nucleotides, had any barcode or primer errors, contained any ambiguous characters, or contained any stretch of >8 homopolymers. Sequences were assigned to their respective samples based on a 10 nucleotide barcode sequence, and were analyzed further using the QIIME pipeline [19]. Briefly, representative OTUs from each set were chosen at a minimum sequence identity of 97% using UClust [38] and aligned using PyNast [39] against the Greengenes database [40]. Multiple alignments then were used to create phylogenies using FastTree [41], and taxonomy was assigned to each OTU using the RDP classifier [42,43]. PCOA was performed based on beta diversity using weighted Unifrac distances [44]. Alpha diversity using the Shannon diversity index [45] also was determined using the QIIME pipeline. Statistical differences in alpha diversity were determined using a two-tailed t-test with Microsoft Excel 2007 (Microsoft Corp., Redman, WA).

### Statistical analysis

To assess whether viromes had significant overlap within or between subjects and sample types, we performed a permutation test based on resampling (10,000 iterations) [46,47,48,49,50]. We simulated the distribution of the fraction of shared virome homologues from 2 different sample types from the group of subjects, excluding intra-subject comparisons. For each set, we computed the summed fraction of shared homologues using 1000 random contigs between different subjects, and from these computed an empirical null distribution of our statistic of interest (the fraction of shared homologues). The simulated statistics within each sample type were referred to the null distribution (the fraction of shared homologues between subjects or sample types), and the *p* value was computed as the fraction of times the simulated statistic for each exceeded the null statistic. For comparisons of intra-subject conservation of homologous viruses, we utilized the same technique with randomly selected intra-subject comparisons to a null distribution of randomly selected inter-subject comparisons. We estimated the *p* value based on the fraction of times the intra-subject statistic exceeded that for the null statistic.

### Homologous virus diversity index

To measure alpha diversity in the viral communities, we utilized a technique termed the Homologous Virus Diversity Index (HVDI) [51]. The technique is based on finding high levels of homology among contigs within viromes that likely belong to the same virus but were placed into separate contigs due to limitations of the assembly process [46]. Virome reads were assembled using 98% identity over a minimum of 50% of the read length using CLC Genomics Workbench 4.65 (CLC bio USA, Cambridge, MA), and the resulting contig spectra utilized as the primary input for the index. The contigs then were subjected to BLASTN analysis against a database of contigs from the same subject, and contigs with high degrees of homology (E-value < 10^−20^) over 50% of the length of the shorter contig were assigned to a network representing a single virus. The spectra from each individual contig assigned to a network were accumulated and those corrected spectra used as inputs for the Shannon Index [45]. Statistical differences in the HVDI within and between sample types were determined using a two-tailed t-test with Microsoft Excel 2007 (Microsoft Corp., Redman, WA).

## Results

### Human subjects and preparation of viromes

We recruited 9 human subjects; four of whom were receiving a 6-week course of intravenous antibiotics, and five as healthy controls (S1 Table). All subjects had no antibiotic exposure for at least 6 months prior to study enrollment. We obtained saliva and fecal samples from each subject on day #3 of antibiotics (time A), week #2 of antibiotics (time B), and at the end of a 6-week course of antibiotics (time C). We collected saliva and fecal samples from control subjects at similar time intervals, however, control subjects were not exposed to any antibiotics during the study.

We isolated and processed viruses from saliva and feces utilizing sequential filtering and cesium chloride density gradient centrifugation according to our previously described protocols [1], and the resulting DNA was subjected to semiconductor sequencing [32]. We sequenced the fecal and salivary viromes from each subject at all time points for a total of 53 viromes (27 from saliva and 26 from feces). We produced 33,090,136 reads from saliva and feces of mean length 213 nucleotides for an average of 3,676,682 reads per subject and 1,272,698 reads per time point (S2 Table). We used BLASTN to compare all viromes to the RDP 16S rRNA database and a human reference genome and found that all were free of 16S rRNA homologues, and relatively few were homologous to human DNA. These data indicated that these oral and fecal viromes were relatively free of contaminating cellular nucleic acids.

### Identification of viruses and viral functions

We assembled virome reads from each subject to construct longer contigs in order to enable more productive searches for homologous sequences. The mean contig length from fecal viromes was 1001±109 nucleotides; for salivary viromes it was 1188±73 nucleotides. Using BLASTX analysis, we identified homologues for each contig against the NCBI NR database. Approximately 30.3 ± 5.0% (range 22.3 to 45.0%) of the virome contigs were homologous to known viruses (S1 Fig), somewhat similar to results found in other studies [1,2,7]; 33.3 ± 5.2% of the fecal viromes and 27.4 ± 2.7% of the salivary viromes had viral homologues. The relative proportions of the viromes with identifiable viral homologues did not differ for saliva or feces based on antibiotic status or length of time on antibiotics. Among the assembled contigs, we identified homologues with a broad array of viral functions, including those involved in virus structure, virulence, and replication. The most common identifiable viral homologues identified in the feces of all subjects were DNA polymerases (17.0 ± 2.8%), integrases/transposases (15.1 ± 2.4%), and hypothetical phage genes (18.0 ± 3.8% of the contigs) (S2 Fig). In saliva there were DNA polymerases (17.9 ± 2.7%), integrases/transposases (15.6 ± 2.2%), helicases (14.4 ± 1.8%), and restriction/modification enzymes (12.3 ± 2.4%) (S3 Fig). The presence of multiple identifiable viral homologues in many of the assembled contigs provides strong support that each sample was highly enriched for bacteriophage.

### Beta diversity in viral communities by body site

**Fig 1:**
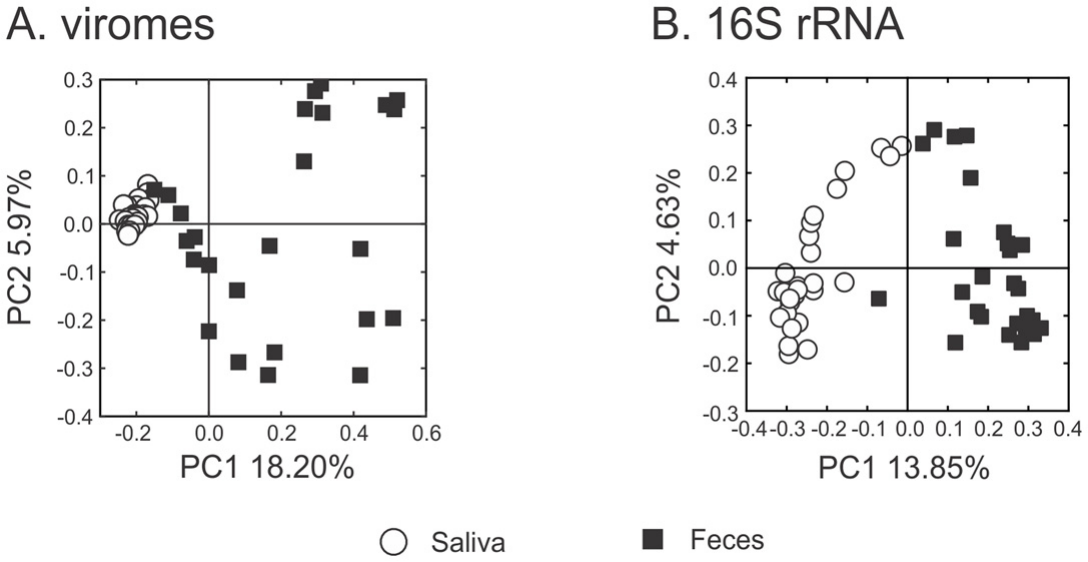
Principal coordinates analysis of beta diversity present in the viromes (Panel A) and the bacterial biota by 16S rRNA (Panel B).

We characterized the viral communities from the feces and saliva of each subject to decipher whether they differed by body site. We examined beta diversity between the viral communities using principal coordinates analysis (PCOA) and observed variation between communities based on the body site examined (Fig 1, Panel A). There was little heterogeneity observed among the salivary viral communities compared to that found among viral contigs within the feces. These findings support that there is greater conservation among the oral viral community than is present in the gut. By using a permutation test [46,47] on contigs shared within and between different body sites, we observed that there was a significant conservation of viral homologues in the mouth, but not in the gut (Table 1).

**Table 1:** Viral homologues within and between subject groups.

	Virome		
	Percentage homologous within a group^a^	Percentage homologous between groups^a^	p-value^b^
Saliva	27.93 ± 4.28^c^	3.97 ± 4.12	**<0.001**
Feces	13.31 ± 8.87	4.06 ± 4.15	0.134
**Feces**			
Controls	16.21 ± 2.62	4.66 ± 4.24	**0.017**
Antibiotics	11.67 ± 8.91	5.10 ± 4.67	0.271
**Saliva**			
Controls	23.38 ± 3.63	27.34 ± 4.56	0.751
Antibiotics	27.86 ± 3.81	28.22 ± 4.59	0.514

^a^Based on the mean of 10,000 iterations. 1,000 random contigs were sampled per iteration.

^b^Empirical p-value based on the fraction of times the estimated percent homologous contigs for each group exceeded that between groups.

^c^± indicates the standard deviation.

We also characterized the membership of the salivary and fecal bacterial communities in the same subjects to determine whether similar trends present in the viral communities were observed in the bacteria. We sequenced the V1-V2 hypervariable segment of 16S rRNA (S3 Table), and similar to trends observed in viral communities, bacterial communities varied according to the body site from which they were derived (Fig 1, Panel B).

### BLASTX putative host profiles of oral and gut viromes

**Fig 2:**
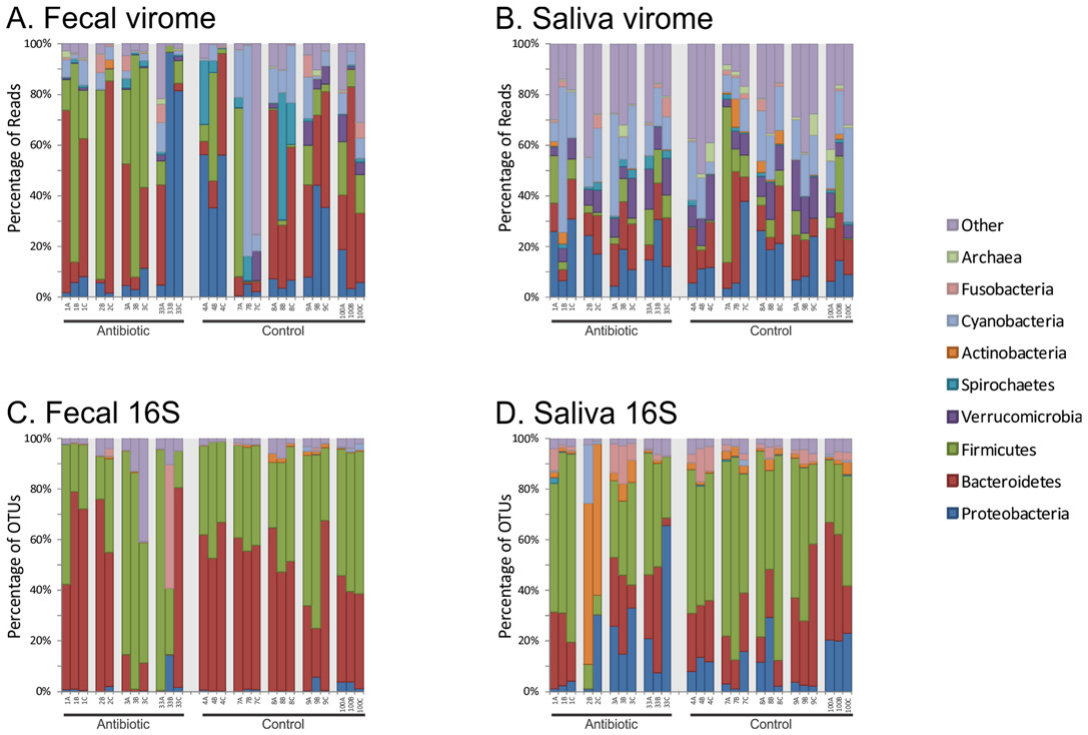
Bar graphs demonstrating proportion of viral reads homologous to phage that infect members of certain bacterial phyla for feces (Panel A) and saliva (Panel B), or the proportion of 16S rRNA sequence reads from certain phyla for feces (Panel C) and saliva (Panel D). The y-axis shows the percentage of reads, and the x-axis represents the different subjects studied. Time A represents day #3, time B represents week #2, and time C represents week #6.

We assessed the putative host profiles by BLASTX to determine whether similar trends may be identified between saliva and feces. We utilized the phylum level taxonomic classification of the bacterial hosts for each viral homologue for comparisons between subjects and body sites. There were numerous homologues to viruses from the phyla Firmicutes, Bacteroidetes, and Proteobacteria among others identified in the feces of each subject (Fig 2, Panel A). There was a significant decrease in the number of homologues to Bacteroidetes in each subject after 2 weeks of antibiotics with a concomitant increase in homologues to Firmicutes (S4 Fig, Panel A). Similar phyla were observed in the saliva of these subjects (Fig 2, Panel B), but no general patterns were observed over time in response to antibiotics (S4 Fig, Panel B). For comparison, we analyzed the bacterial taxonomies using 16S rRNA and found that there was some taxonomic variation in those subjects on antibiotics (Fig 2, Panels C and D). Subjects #3 and #33 had decreases in Bacteroidetes with concomitant increases in Firmicutes in the feces. In the saliva, subject #2 had substantial increases in Actinobacteria.

**Fig 3:**
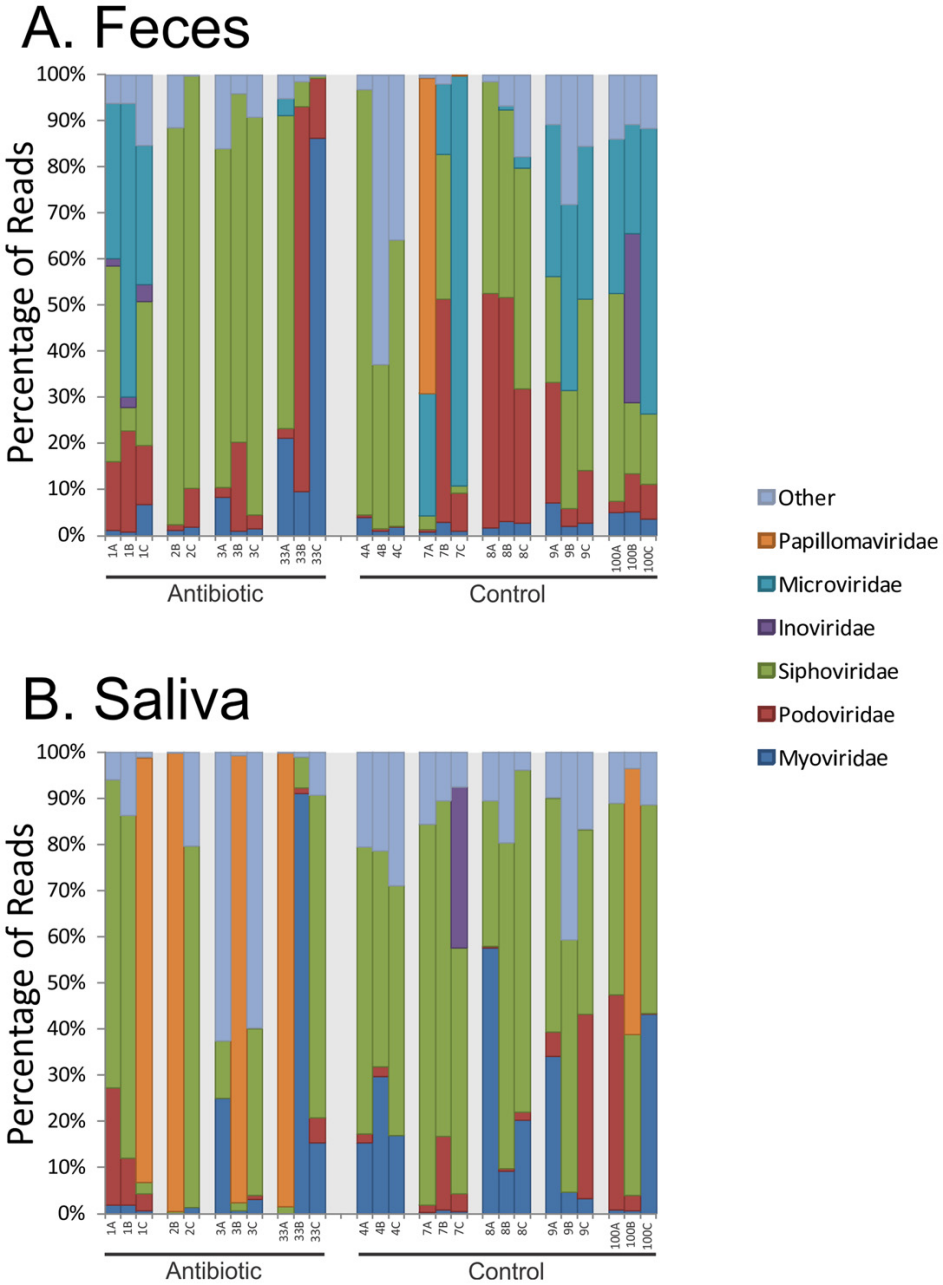
Bar graphs demonstrating proportion of viral reads homologous to different virus families for feces (Panel A) and saliva (Panel B). The y-axis shows the percentage of reads, and the x-axis represents the different subjects studied. Time A represents day #3, time B represents week #2, and time C represents week #6.

We also characterized the virus families identified in all subjects over time to determine whether differences existed between the oral cavity and the gut, and whether there was evidence of a response to antibiotics. We found viruses homologous to each different Caudovirus family, including Myoviridae, Podoviridae, and Siphoviridae (Fig 3, Panels A and B). Siphoviruses generally have temperate lifestyles and represented the majority of the virus families identified. Their presence along with the high proportion of integrases/transposases identified (S2 and S3 Figs), suggests that the majority of the viruses in both the oral cavity and gut had temperate lifestyles. We also identified single stranded DNA viruses, including families Inoviridae and Microviridae. The family Microviridae was more common in the gut virome (Fig 3, Panel A), while few were identified in the oral cavity (Fig 3,Panel B). Among the other viruses identified with homologues present in the gut and oral viromes were herpesviruses, phycodnaviruses, poxviruses, mimiviruses, and baculoviruses. Interestingly, in all 4 subjects on antibiotics, the majority of their oral viral populations were papillomaviruses at some point during their antibiotic therapy, compared to only 1 of the 5 controls that had a significant number of papillomaviruses during the study. We previously identified papillomaviruses in the human urine virome [51], and their high proportions in the oral viromes of subjects taking antibiotics in this study suggests that the use of antibiotics may be associated with their increased production.

### Viral community diversity in response to antibiotics

**Fig 4:**
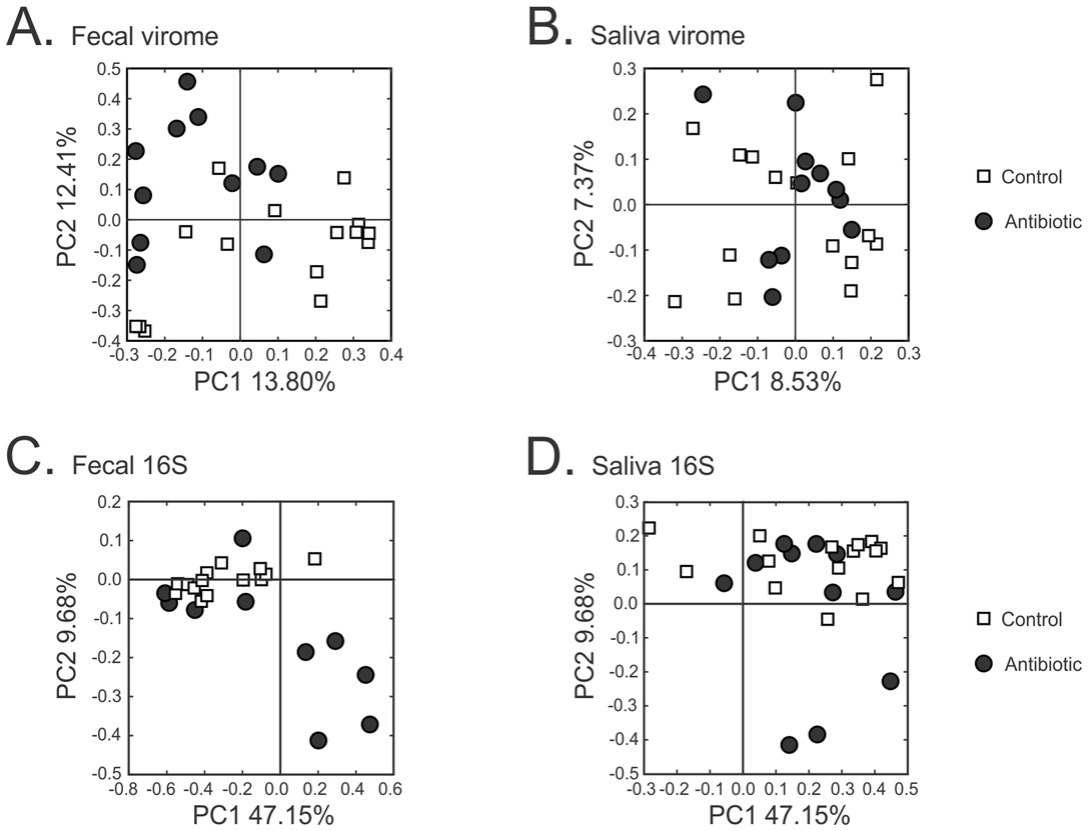
Principal coordinates analysis of beta diversity among the viromes of each subject and time point. Panel A represents fecal viromes, Panel B represents salivary viromes, Panel C represents fecal 16S rRNA, and Panel D represents salivary 16S rRNA.

Because the pattern of BLASTX homologues suggested that the viral communities may not be altered significantly in response to antibiotics, we compared the beta diversity among the viromes of those subjects on antibiotics compared to controls. There was some variation observable by PCOA specific to fecal viromes, but only a small proportion could be explained by antibiotic status (Fig 4, Panel A). There were no identifiable patterns observed in the saliva of subjects on antibiotics compared to controls (Fig 4, Panel B). Using a permutation test [46,47], we found that a small but significant proportion of the fecal viromes was conserved among the control subjects (p = 0.017), but not for those subjects on antibiotics (p = 0.271) (Table 1). There were no statistical differences identified in saliva viromes based on antibiotic status. No observable differences could be identified among the bacterial biota based on antibiotic status in saliva or feces (Fig 4, Panels C and D), but there generally was less heterogeneity among the fecal bacterial biota in the control subjects compared to those on antibiotics.

**Fig 5:**
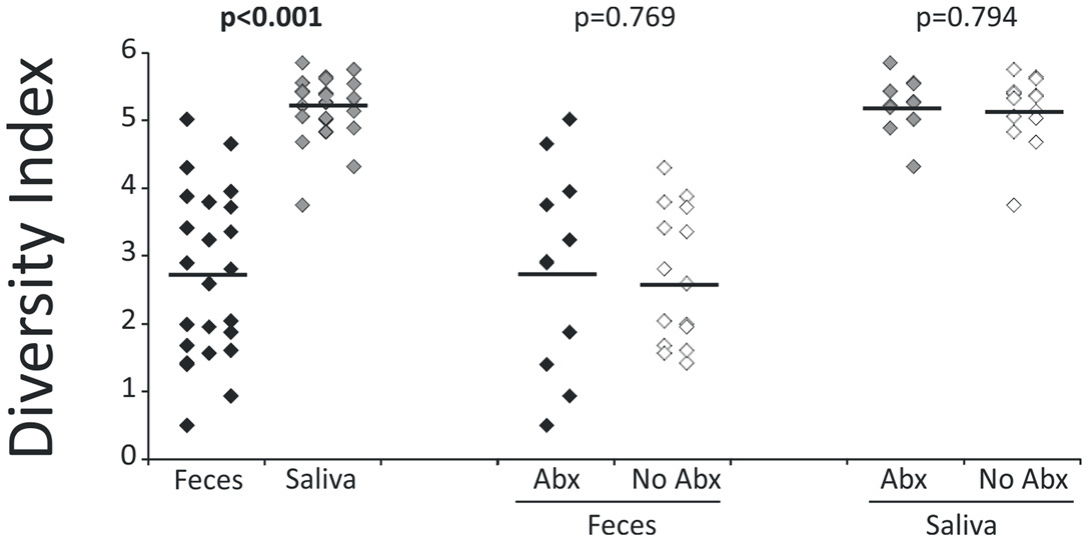
Plot of the Homologous Virus Diversity Index for all subjects. The y-axis represents the diversity index, and the x-axis from left to right represents the feces and saliva from all subjects, the feces from subjects under antibiotic treatment and controls, and the saliva of subjects under antibiotic treatment and their controls. P-values are represented above each diagram, and values ≤0.05 are represented in bold.

We previously developed tools for characterizing viral communities to determine the adequacy of sequencing efforts and to compare diversity between viral communities [51]. The Homologous Virus Diversity Index (HVDI) is based upon the Shannon Index [45] and utilizes modified contig spectra to substitute for community structures. We used the results of the HVDI to investigate whether the alpha diversity of viruses in the feces and saliva of the subjects was significantly different. We found that salivary viromes had a much more diverse population of viruses compared to fecal viromes consistently among most subjects and samples (Fig 5; p<0.001). We also compared alpha diversity in viromes from control subjects with those from subjects on antibiotics. When we compared the diversity of the viral communities in the feces based on antibiotic status, there was no measurable difference between groups (Fig 5). There also was substantial heterogeneity in the fecal communities regardless of antibiotic status compared with saliva. These results indicate that antibiotic therapy did not have a significant impact upon the diversity of viruses in the gut in this subject population. Similar results were demonstrated for the viral communities in saliva. We also examined alpha diversity for the bacterial biota as determined by the Shannon Index, and found that there were significant differences (p = 0.001 for feces and p = 0.018 for saliva) in diversity in subjects on antibiotics (S5 Fig). Our results on viral diversity suggest that oral and fecal viral population structures were largely not affected by antibiotics, though bacterial biota were.

### Subject specificity in fecal and salivary viromes

We tested whether the fecal and salivary viromes in each subject were reflective of their host environment despite the antibiotic perturbations. For control subjects, virome contents were reflective of the individual host environment in both saliva and feces (Table 2). This same trend also was observed in the saliva of those subjects on antibiotics, which indicates that the use of antibiotics did not sufficiently modify the contents of the oral viromes to alter their subject specific features. Several of the fecal viromes also demonstrated subject-specific features despite the use of antibiotics, although not all the differences observed were statistically significant.

**Table 2:** Fecal and salivary viral homologues within and between subject groups.

	Virome		
	Percentage homologous within a group^a^	Percentage homologous between groups^a^	p-value^b^
**Feces**			
**Antibiotic**			
ELA1	47.64 ± 4.74^c^	6.33 ± 4.42	**<0.001**
ELA2	0.88 ± 0.29	4.39 ± 7.70	0.456
ELA3	14.51 ± 14.68	6.07 ± 6.93	0.340
ELA33	17.83 ± 10.19	2.64 ± 2.28	**0.040**
**Control**			
ELA4	69.21 ± 2.26	17.83 ± 10.95	**<0.001**
ELA7	42.38 ± 5.40	5.87 ± 4.70	**<0.001**
ELA8	44.25 ± 3.02	7.07 ± 4.79	**<0.001**
ELA9	59.84 ± 1.51	4.55 ± 3.53	**<0.001**
ELA100	56.74 ± 2.39	4.72 ± 4.13	**<0.001**
**Saliva**			
**Antibiotic**			
ELA1	30.25 ± 4.87	7.53 ± 2.15	**<0.001**
ELA2	24.88 ± 1.13	7.94 ± 1.93	**<0.001**
ELA3	27.35 ± 5.17	9.60 ± 3.31	**0.001**
ELA33	36.13 ± 1.64	10.68 ± 2.76	**<0.001**
**Control**			
ELA4	30.47 ± 3.43	9.68 ± 2.47	**<0.001**
ELA7	41.81 ± 2.17	8.71 ± 2.55	**<0.001**
ELA8	33.54 ± 1.76	9.53 ± 2.34	**<0.001**
ELA9	29.49 ± 1.88	8.40 ± 2.31	**<0.001**
ELA100	32.97 ± 2.36	6.81 ± 2.31	**<0.001**

^a^Based on the mean of 10,000 iterations. 1,000 random contigs were sampled per iteration.

^b^Empirical p-value based on the fraction of times the estimated percent homologous contigs for each group exceeded that between groups.

^c^± indicates the standard deviation.

### Antibiotic resistance genes in fecal and salivary viromes

**Fig 6:**
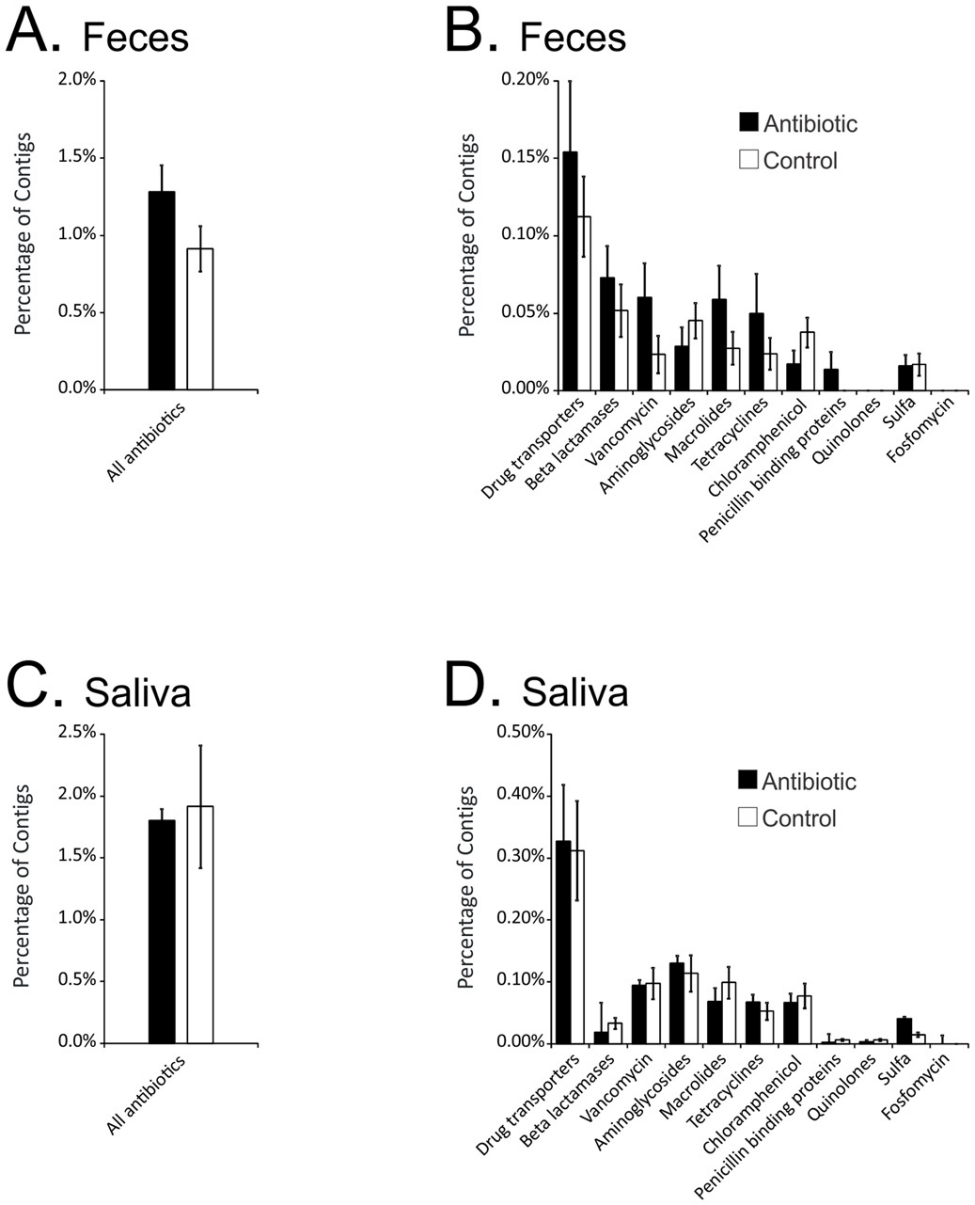
Plots of the percentage (±standard error) of contigs with ORFs homologous to genes involved in antibiotic resistance in feces (Panels A and B) and salivary viromes (Panel C and D). The percentage of contigs is demonstrated on the y-axis, and the class of antibiotic resistance is shown on the x-axis.

The subjects exposed to antibiotics in this study were all treated with broad spectrum IV antibiotics, and we hypothesized that the constant selective pressure would result in an increase in the reservoir of antibiotic resistance genes in the human viral communities as previously had been shown for murine viral communities [23]. We compared the proportions of the virome contigs homologous to antibiotic resistance genes in the Comprehensive Antibiotic Resistance Database (CARD) [36] by BLASTX analysis. Fecal viromes from persons on antibiotics contained an elevated abundance of antibiotic resistance gene homologues compared to viral communities from controls (Fig 6, Panel A). Interestingly, homologues to genes involved in resistance to beta lactams, vancomycin, macrolides, tetracyclines, penicillin binding proteins, and multi-drug transporters were all increased in subjects on antibiotics (Fig 6, Panel B), however, none of the differences met statistical significance. While a proportion of the salivary viromes were homologous to antibiotic resistance genes, there was no relationship between antibiotic resistance gene relative abundance and antibiotic treatment status (Fig 6, Panels C and D).

## Discussion

Viruses are essential players in microbial ecology, but their community structures and responses to perturbations in humans are not well understood. Here we used metagenomic techniques to characterize human viral communities and to illustrate how their ecology differs on different body surfaces. While it has been well described that the bacterial biota of the gut and the oral cavity differ [52], this trend has not been thoroughly examined in viral communities. Because the oral cavity is continuous with the gastrointestinal tract, it is likely that there are some shared viruses, such as crAssphage, which was recently identified from both body sites [53]. While it is important to note that only approximately a third of viral sequences were able to be characterized and analyzed, two thirds remain uncharacterized and uncategorized. The analysis presented here supports that there are significant differences in the viromes of the oral and gastrointestinal tracts. Most of the viruses identified on both body surfaces were bacteriophage; however, the methods used in this study did not include RNA viruses and isolated viruses according to their densities, which could have reduced the number of eukaryotic viruses identified. Our findings of papillomaviruses in the saliva of the subjects taking antibiotics suggests that their expression could be associated with antibiotic use, but a larger cohort would be needed determine whether a true association exists. We also used multiple displacement amplification (MDA) to amplify viral material prior to sequencing. MDA can introduce amplification biases into viromes [17,18], but less than other prior methods [54]. The same amplification process was applied to each saliva and fecal sample, so the biases likely were equally distributed among samples and trends over time between study groups. Bacteria in the human gut microbiome are known to change with age [55], and the significant difference in ages of the subjects in this study could confound results. The affects of age on the human virome have not yet been studied; however, because many of the viruses identified infect bacteria, they are likely to show some association subject age.

Previous studies have determined that phage in the oral cavity [46] and the gut [56] may persist over time, which suggests that viruses on these body surfaces have developed a dynamic equilibrium with their bacteria hosts that allows for both virus and host to persist. The fact that there was significant subject specificity to the viral communities in both the feces and saliva of this cohort (Table 2) indicated that some of the viruses studied likely persisted over time. Their persistence despite antibiotic perturbations implied that either their cellular hosts were not eradicated or that the changes in the relative abundances of their hosts were not sufficient to significantly alter the ecology of their viral communities. Not all time points in all the subjects on antibiotics showed significant increases in the reservoir of antibiotic resistance, but the trend among all subjects was clear in the gut. We have previously found that not all time points will accurately represent the virome of an individual subject [1], but when subjects are sampled on finer time scales their trends become clear [46]. Future studies of the antibiotic resistance reservoir in humans should be performed on finer time scales.

As has previously been shown in mice in response to antibiotic perturbations [23], our data shows a consistent but not statistically significant increase in the gut reservoir of antibiotic resistance in response to antibiotics (Fig 6). The apparent increase in the relative abundance of antibiotic resistance gene homologues in fecal viromes suggests that human gut viral communities are sensitive responders to their environment, and could potentially play a role in promoting antibiotic resistance in their bacterial hosts, but this requires future study with greater power to test for significance. It remains to be determined whether this increase represents new viruses entering the community or whether it is simply due to increases in the relative abundances of viruses already present at low levels or as latent reservoirs. The depth of sequencing of the viral communities in this study likely is insufficient to adequately determine whether the increase in antibiotic resistance may be due to increases in the representation of low abundance viruses. Interestingly, there was an observed increase in antibiotic resistance in the viral community in the gut, but there was no concomitant response observed in the saliva. There were numerous viruses in the saliva that carried antibiotic resistance homologues, but their lack of response to antibiotics suggests that the host bacteria for these viruses may be relatively unperturbed by antibiotic administration.

Measuring the diversity of viral communities accurately has relied upon the use of contig spectra from the metagenomic assembly process as surrogates for population structures [22] among other techniques [21]. Our prior work has shown that limitations in the assembly process can lead to overestimates of viral diversity [46]. We utilized a technique, HVDI, where we assigned viral contigs with high degrees of homology to construct networks of contigs likely belonging to the same virus and utilized the corrected contig spectra as input for diversity measures [51]. This technique demonstrated that there were significant differences in the population structures of viruses in saliva and feces (Fig 5). That we found higher levels of diversity in the oral viral communities of these subjects than we found in their feces fits with the greater diversity that also was observed in their host bacteria (S5 Fig). In a prior study comparing fecal and salivary viromes, the fecal viromes were relatively homogenous compared to salivary viromes [1]. Those fecal viromes were derived from twins and their mothers [3], which likely accounts for the significant conservation observed amongst those fecal viromes. The heterogeneity amongst the fecal viromes found in this study may be related to the different antibiotics taken by the subjects (S1 Table). Virus-like particles in the feces generally outnumber those found in saliva [57], so the increased diversity in saliva found in this study was not due to a greater abundance of viruses. Viral community diversity was not affected by the use of antibiotics on either body surface, which suggests that new viruses replaced departed viruses whose hosts were eradicated by antibiotics or that the changes in the relative abundance of host bacteria are not sufficient to determine viral ecology. Further studies, potentially using model ecosystems [58,59] will be necessary to help decipher the mechanism by which viral diversity remains a stable component of the ecosystem while bacterial diversity is lessened.

The ecology of viral communities is an important consideration as we continue to explore the effects of perturbations to indigenous microbiota in human health and disease. Viruses may be significant drivers of bacterial diversity [60,61], and the fact that their communities are heavily populated on human body surfaces makes it difficult to discern their role. Our data help to elucidate ecological features of human viral communities and showed that there are significant differences between oral and gut communities based on their membership, diversity, and responses to antibiotic perturbations. We hypothesize that the relative stability of the oral viral community may be due to the highly dynamic nature of the oral environment with constant exposure to multiple perturbations such as food, beverages, and dental hygiene, where the community is capable of rapidly repopulating a large portion of its viruses. This is in contrast to the colon where feces often remain for 24–48 hours or more, facing prolonged exposure to antibiotics prior to evacuation. The prolonged exposure to antibiotics may be responsible for the observed rise in antibiotic resistance genes identified in fecal viromes in the setting of antibiotic exposure compared to saliva. We believe that understanding the role of viral communities in driving bacterial diversity and potentially in the transmission of antibiotic resistance in the diverse human oral and gut ecosystem highlights the need for the development of cultured viral ecosystems that can model the dynamic interactions of viruses with their hosts in humans.

## Supporting Information

S1 Fig Bar graph of the percentage of contigs with viral homologues in the NR database from the feces (Panel A) and the saliva (Panel B) of all subjects. The percentage of contigs with viral homologues is shown on the y-axis and the subjects and relevant time points are shown on the x-axis. Subjects on antibiotics are demonstrated by black bars and control subjects are represented by white bars. Time A represents day #3, time B represents week #2, and time C represents week #6.
(PDF)

S2 Fig Bar graph of the percentage of contigs (± standard error) with viral homologues in the NR database from the feces of all subjects. The annotation of each homologue is shown on the x-axis and the y-axis represents the percentage of contigs.
(PDF)

S3 Fig Bar graph of the percentage of contigs (± standard error) with viral homologues in the NR database from the saliva of all subjects. The annotation of each homologue is shown on the x-axis and the y-axis represents the percentage of contigs.
(PDF)

S4 Fig Charts representing the proportion of virome reads (±standard error) with BLASTX homology to phage with hosts from different bacterial phyla (Panels A and B) or the proportion of the bacterial biota belonging to certain phyla (Panels C and D). Panels A and B represent virome BLASTX hits and Panels C and D represent 16S rRNA taxonomic assignments. Panels A and C represent fecal microbiota and Panels B and D represent salivary microbiota.
(PDF)

S5 Fig Shannon diversity index values (±standard error) for fecal and salivary bacterial biota based on 16S rRNA. Subjects on antibiotics are represented by black bars and control subjects are represented by white bars. P-values are represented above each diagram, and values ≤0.05 are represented in bold.
(PDF)

S1 Table Study subjects. (DOCX)

S2 Table Fecal and saliva virome reads. (DOCX)

S3 Table Fecal and saliva 16S rRNA reads. (DOCX)
