# Supplementary material for: Effects of Long Term Antibiotic Therapy on Human Oral and Fecal Viromes
Source: PLoS One. 2015 Aug 26;10(8):e0134941. doi: 10.1371/journal.pone.0134941 (PMC4550281; doi:10.1371/journal.pone.0134941)
Supplement: S1 Fig — The percentage of contigs with viral homologues is shown on the y-axis and the subjects and relevant time points are shown on the x-axis. Subjects on antibiotics are demonstrated by black bars and control subjects are represented by white bars. Time A represents day #3, time B represents week #2, and time C represents week #6. (PDF) [file pone.0134941.s001.pdf]

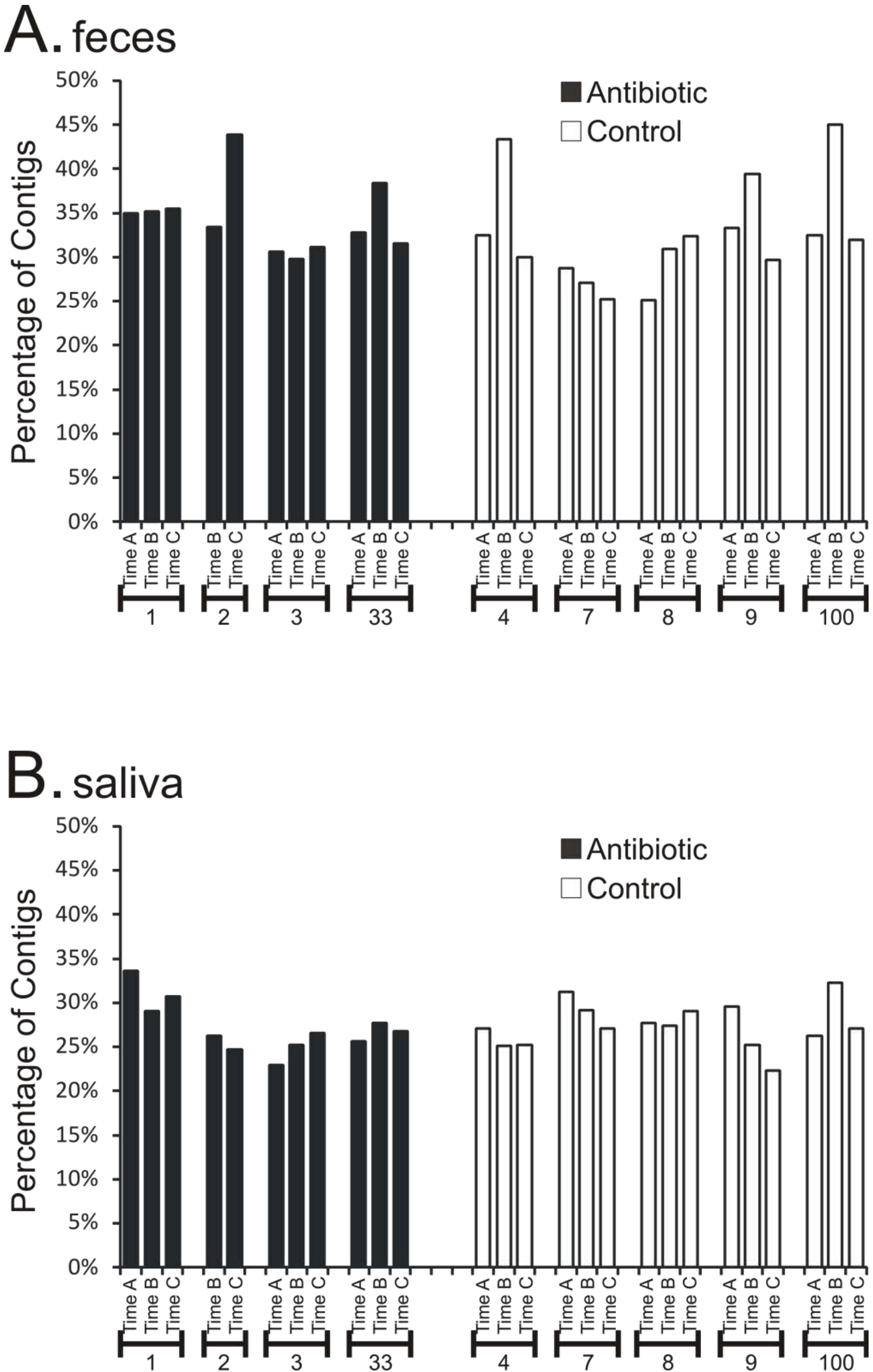

S1 Fig.: Bar graph of the percentage of contigs with viral homologues in the NR database from the feces (Panel A) and the saliva (Panel B) of all subjects. The percentage of contigs with viral homologues is shown on the y-axis and the subjects and relevant time points are shown on the x-axis. Time A represents day #3, time B represents week #2, and time C represents week #6.
