# Supplementary material for: Effects of Long Term Antibiotic Therapy on Human Oral and Fecal Viromes
Source: PLoS One. 2015 Aug 26;10(8):e0134941. doi: 10.1371/journal.pone.0134941 (PMC4550281; doi:10.1371/journal.pone.0134941)
Supplement: S2 Fig — The annotation of each homologue is shown on the x-axis and the y-axis represents the percentage of contigs. (PDF) [file pone.0134941.s002.pdf]

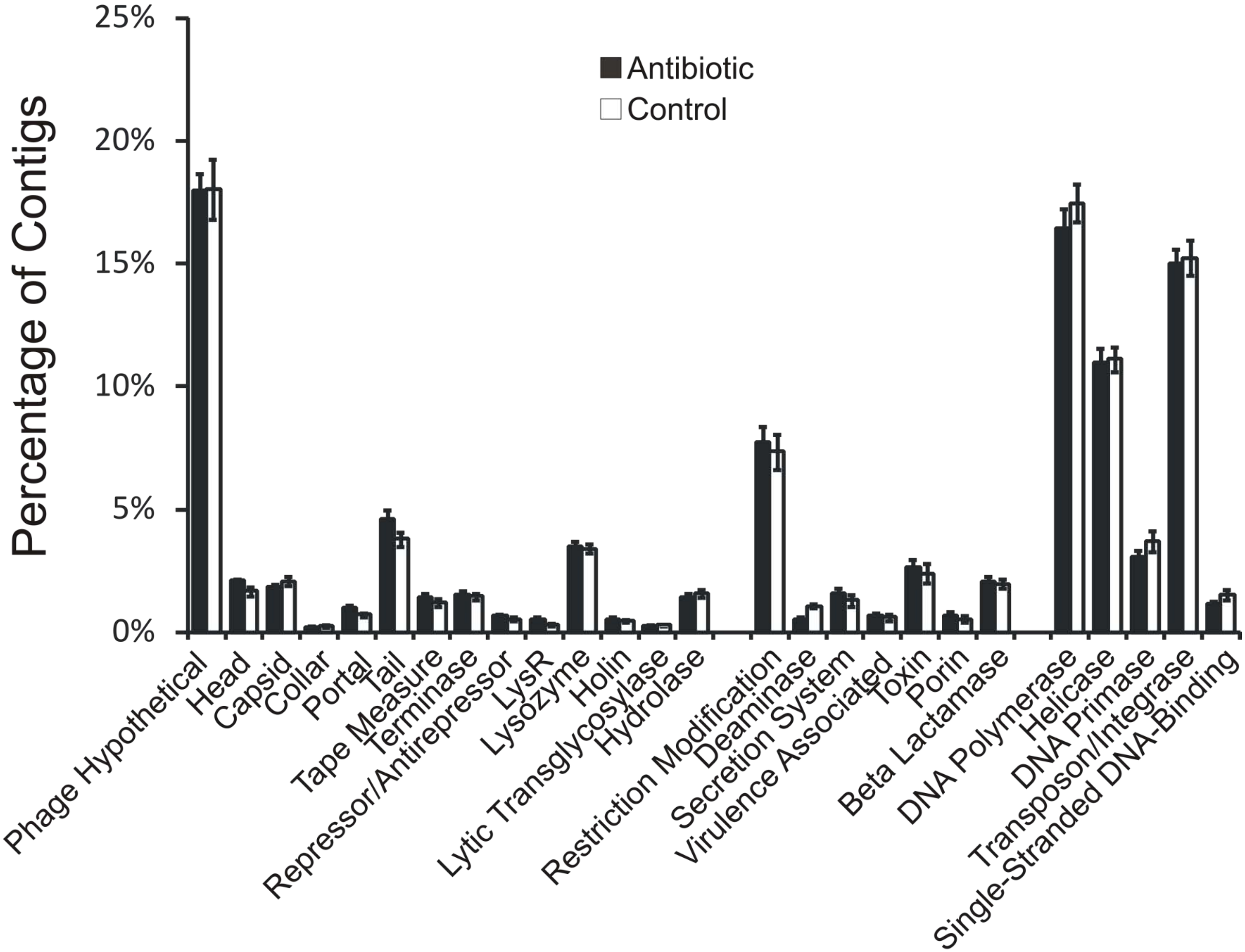

S2 Fig.: Bar graph of the percentage of contigs (± standard error) with viral homologues in the NR database from the feces of all subjects. The annotation of each homologue is shown on the x-axis and the y-axis represents the percentage of contigs.
