# Supplementary material for: Effects of Long Term Antibiotic Therapy on Human Oral and Fecal Viromes
Source: PLoS One. 2015 Aug 26;10(8):e0134941. doi: 10.1371/journal.pone.0134941 (PMC4550281; doi:10.1371/journal.pone.0134941)
Supplement: S4 Fig — Panels A and B represent virome BLASTX hits and Panels C and D represent 16S rRNA taxonomic assignments. Panels A and C represent fecal microbiota and Panels B and D represent salivary microbiota. (PDF) [file pone.0134941.s004.pdf]

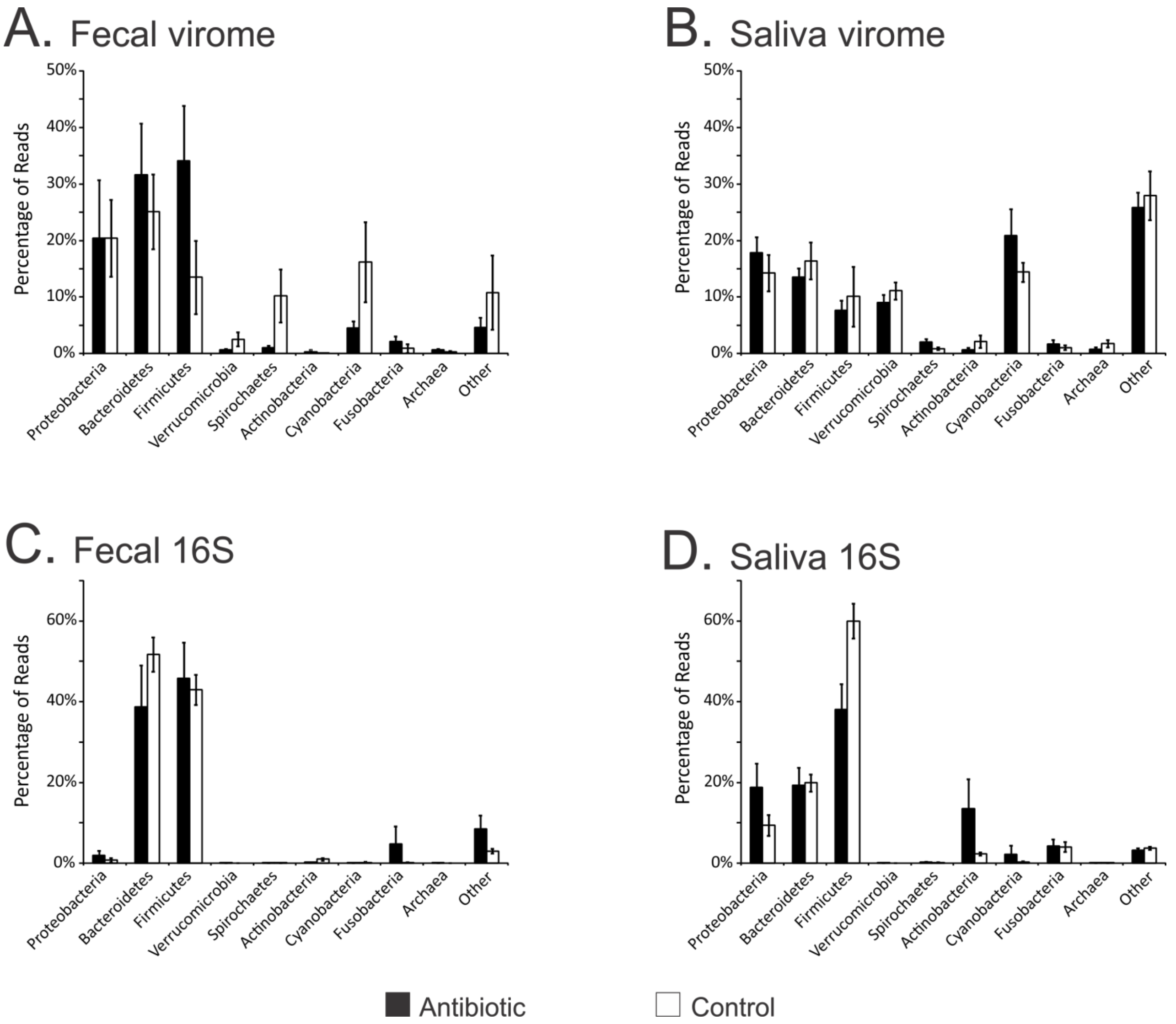

S4 Fig.: Charts representing the proportion of virome reads (±standard error) with BLASTX homology to phage with hosts from different bacterial phyla (Panels A and B) or the proportion of the bacterial biota belonging to certain phyla (Panels C and D). Panels A and B represent virome BLASTX hits and Panels C and D represent 16S rRNA taxonomic assignments. Panels A and C represent fecal microbiota and Panels B and D represent salivary microbiota.
