# Supplementary material for: Effects of Long Term Antibiotic Therapy on Human Oral and Fecal Viromes
Source: PLoS One. 2015 Aug 26;10(8):e0134941. doi: 10.1371/journal.pone.0134941 (PMC4550281; doi:10.1371/journal.pone.0134941)
Supplement: S5 Fig — Subjects on antibiotics are represented by black bars and control subjects are represented by white bars. P-values are represented above each diagram, and values ≤0.05 are represented in bold. (PDF) [file pone.0134941.s005.pdf]

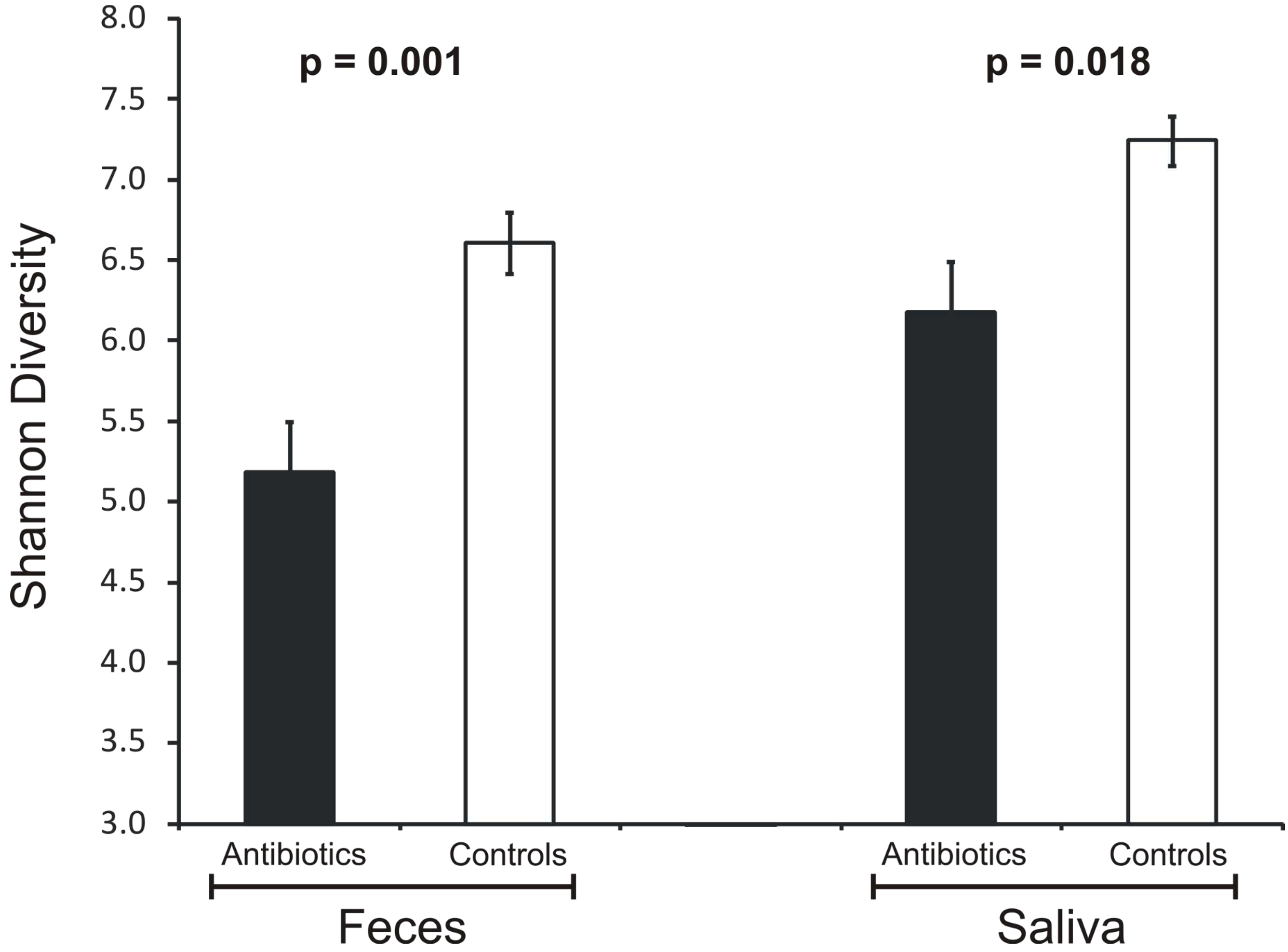

S5 Fig: Shannon diversity index values (±standard error) for fecal and salivary bacterial biota based on 16S rRNA. P-values are represented above each diagram, and values ≤0.05 are represented in bold.
