# Supplementary material for: Effects of Long Term Antibiotic Therapy on Human Oral and Fecal Viromes
Source: PLoS One. 2015 Aug 26;10(8):e0134941. doi: 10.1371/journal.pone.0134941 (PMC4550281; doi:10.1371/journal.pone.0134941)
Supplement: S1 Table — (DOCX) [file pone.0134941.s006.docx]

**S1 Table.** Study Subjects

| **Subject** | **Age** | | **Ethnicity** | **Sex** | **Diagnosis** | **Antibiotics** | **Pathogen** |
| --- | --- | --- | --- | --- | --- | --- | --- |
| **Subjects on antibiotics** | | | | | |  |  |
| ELA1 | | 73 | Caucasian | Male | Bone infection | Cefazolin and trimethoprim/sulfamethaxole | MSSA^a^ |
| ELA2 | | 66 | Caucasian | Male | Bone infection | Vancomycin and meropenem | MRSA^b^ and Pseudomonas aeruginosa |
| ELA3 | | 62 | Caucasian | Male | Bone infection | Vancomycin, daptomycin and ceftazidime | Coagulase negative staphylococcus |
| ELA33 | | 69 | Caucasian | Female | Brain abscess | Vancomycin and rifampin | MRSA and Enterococcus faecalis |
|  | | | | | |  |  |
| **Control subjects** | | | | | |  |  |
| ELA4 | | 25 | Asian | Male | Healthy | None | None |
| ELA7 | | 25 | Caucasian | Male | Healthy | None | None |
| ELA8 | | 51 | Latino | Female | Healthy | None | None |
| ELA9 | | 34 | African-American | Male | Healthy | None | None |
| ELA100 | | 32 | Asian | Female | Healthy | None | None |

^a^Methicillin Sensitive Staphylococcus aureus

^b^Methicillin Resistant Staphylococcus aureus
