# Supplementary material for: Effects of Long Term Antibiotic Therapy on Human Oral and Fecal Viromes
Source: PLoS One. 2015 Aug 26;10(8):e0134941. doi: 10.1371/journal.pone.0134941 (PMC4550281; doi:10.1371/journal.pone.0134941)
Supplement: S2 Table — (DOCX) [file pone.0134941.s007.docx]

**S2A Table.** Fecal virome reads and contigs

| **Reads** | | | | | **Contigs** | | | |
| --- | --- | --- | --- | --- | --- | --- | --- | --- |
| **Sample** | **Final Reads^a^** | **Length** | **No.**  **16s^b^** | **No. Human^c^** | **Number** | **Average Length** | **G+C Content** | **N50** |
| **Feces** |  |  |  |  |  |  |  |  |
| **Antibiotics** |  |  |  |  |  |  |  |  |
| ELA1A^d^ | 778033 | 218 | 0 | 0 | 3674 | 955 | 43.23% | 1533 |
| ELA1B^e^ | 484525 | 209 | 0 | 0 | 2524 | 826 | 44.27% | 1076 |
| ELA1C^f^ | 869569 | 219 | 0 | 1 | 4919 | 1148 | 43.69% | 2284 |
| ELA2B | 481023 | 214 | 0 | 1 | 1590 | 1075 | 42.86% | 1926 |
| ELA2C | 498831 | 211 | 0 | 1 | 549 | 845 | 41.92% | 1065 |
| ELA3A | 646903 | 214 | 0 | 6 | 4224 | 994 | 42.68% | 1605 |
| ELA3B | 668496 | 215 | 0 | 0 | 664 | 799 | 45.26% | 1142 |
| ELA3C | 841975 | 215 | 0 | 2 | 3913 | 1011 | 43.95% | 1690 |
| ELA33A | 804021 | 215 | 0 | 1 | 4111 | 1134 | 40.49% | 2132 |
| ELA33B | 772493 | 225 | 0 | 0 | 768 | 951 | 45.49% | 1458 |
| ELA33C | 714861 | 218 | 0 | 1 | 2541 | 1002 | 42.16% | 1570 |
| **Controls** |  |  |  |  |  |  |  |  |
| ELA4A | 940188 | 223 | 0 | 1 | 860 | 1094 | 42.19% | 2319 |
| ELA4B | 1152615 | 217 | 0 | 4 | 791 | 1145 | 45.88% |  |
| ELA4C | 823709 | 217 | 0 | 0 | 902 | 1069 | 42.5% | 1959 |
| ELA7A | 638728 | 207 | 0 | 0 | 1798 | 920 | 38.93% | 1359 |
| ELA7B | 526780 | 212 | 0 | 0 | 1419 | 898 | 39.76% | 1192 |
| ELA7C | 560889 | 214 | 0 | 0 | 1175 | 908 | 38.69% | 1253 |
| ELA8A | 614979 | 212 | 0 | 1 | 2524 | 1073 | 41.04% | 1857 |
| ELA8B | 532194 | 215 | 0 | 2 | 2594 | 984 | 45.58% | 1580 |
| ELA8C | 750625 | 215 | 0 | 1 | 2112 | 830 | 40.82% | 1045 |
| ELA9A | 766912 | 204 | 0 | 0 | 3651 | 949 | 40.23% | 1395 |
| ELA9B | 598253 | 224 | 0 | 0 | 1853 | 1122 | 39.64% | 2081 |
| ELA9C | 788109 | 209 | 0 | 0 | 3390 | 1031 | 39.34% | 1640 |
| ELA100A | 786942 | 210 | 0 | 2 | 2567 | 1195 | 40% | 2496 |
| ELA100B | 1030895 | 216 | 0 | 1 | 4570 | 1040 | 41.32% | 1654 |
| ELA100C | 878425 | 214 | 0 | 0 | 2188 | 1032 | 38.83% | 1915 |

^a^Final number of reads after trimming and removal of reads with long homopolymers

^b^Based on BLASTN analysis (Escore < 10^-5^) of a composite 16S rRNA database including the full RDP, Greengenes, NCBI, and Silva databases

^c^Based on BLASTN analysis (Escore < 10^-5^) of NCBI human reference genome assemblies

^d^represents the Day 3 time point, ^e^represents the 2 Week time point, ^f^represents the 6 week time point

**S2B Table.** Saliva virome reads and contigs

| **Reads** | | | | | **Contigs** | | | |
| --- | --- | --- | --- | --- | --- | --- | --- | --- |
| **Sample** | **Final Reads^a^** | **Length** | **No.**  **16s^b^** | **No. Human^c^** | **Number** | **Average Length** | **G+C Content** | **N50** |
| **Saliva** |  |  |  |  |  |  |  |  |
| **Antibiotics** |  |  |  |  |  |  |  |  |
| ELA1A^d^ | 419184 | 216 | 0 | 3 | 4198 | 1117 | 41.3% | 1873 |
| ELA1B^e^ | 551727 | 215 | 0 | 0 | 3165 | 1118 | 41.74% | 1810 |
| ELA1C^f^ | 535817 | 213 | 0 | 2 | 3206 | 1194 | 41.1% | 2092 |
| ELA2B | 525960 | 212 | 0 | 1 | 3885 | 1168 | 44.15% | 2141 |
| ELA2C | 476491 | 206 | 0 | 2 | 2659 | 1157 | 42.17% | 2188 |
| ELA3A | 537422 | 201 | 0 | 0 | 2767 | 1199 | 41.19% | 2238 |
| ELA3B | 483638 | 204 | 0 | 0 | 2407 | 1175 | 41.19% | 2339 |
| ELA3C | 407835 | 203 | 0 | 17 | 3138 | 1132 | 43.38% | 2085 |
| ELA33A | 487584 | 204 | 0 | 0 | 2577 | 1236 | 41.51% | 2508 |
| ELA33B | 694385 | 198 | 0 | 1 | 2715 | 1349 | 40.94% | 1964 |
| ELA33C | 440267 | 203 | 0 | 0 | 3234 | 1167 | 42.47% | 1917 |
| **Controls** |  |  |  |  |  |  |  |  |
| ELA4A | 589550 | 213 | 0 | 0 | 3363 | 1188 | 42.82% | 2281 |
| ELA4B | 706672 | 210 | 0 | 4 | 3591 | 1252 | 43.63% | 2314 |
| ELA4C | 603175 | 217 | 0 | 1 | 2944 | 1293 | 43.27% | 2558 |
| ELA7A | 670284 | 220 | 0 | 0 | 2920 | 1177 | 41.01% | 2174 |
| ELA7B | 572484 | 218 | 0 | 1 | 2969 | 1209 | 42.27% | 2289 |
| ELA7C | 553548 | 215 | 0 | 2 | 2908 | 1242 | 41.74% | 2311 |
| ELA8A | 568636 | 217 | 0 | 3 | 3577 | 1148 | 40.61% | 2086 |
| ELA8B | 637037 | 216 | 0 | 0 | 3236 | 1204 | 42.39% | 2375 |
| ELA8C | 482229 | 217 | 0 | 0 | 3091 | 1199 | 41.25% | 2311 |
| ELA9A | 434613 | 220 | 0 | 0 | 2805 | 1380 | 42.28% | 3067 |
| ELA9B | 518883 | 207 | 0 | 1 | 3204 | 1103 | 43.05% | 1848 |
| ELA9C | 433965 | 208 | 0 | 0 | 2784 | 1115 | 41.06% | 1822 |
| ELA100A | 674477 | 214 | 0 | 0 | 4164 | 1155 | 44.22% | 2208 |
| ELA100B | 549595 | 206 | 0 | 1 | 4115 | 1061 | 42.93% | 1738 |
| ELA100C | 583705 | 202 | 0 | 0 | 3395 | 1155 | 42.6% | 2056 |

^a^Final number of reads after trimming and removal of reads with long homopolymers

^b^Based on BLASTN analysis (Escore < 10^-5^) of a composite 16S rRNA database including the full RDP, Greengenes, NCBI, and Silva databases

^c^Based on BLASTN analysis (Escore < 10^-5^) of NCBI human reference genome assemblies

^d^represents the Day 3 time point, ^e^represents the 2 Week time point, ^f^represents the 6 week time point
