# Supplementary material for: Effects of Long Term Antibiotic Therapy on Human Oral and Fecal Viromes
Source: PLoS One. 2015 Aug 26;10(8):e0134941. doi: 10.1371/journal.pone.0134941 (PMC4550281; doi:10.1371/journal.pone.0134941)
Supplement: S3 Table — (DOCX) [file pone.0134941.s008.docx]

**S3A Table.** Fecal 16S rRNA reads

| Sample | Number of Reads | Trimmed or removed^a^ | Final Read Count | Mean Length |
| --- | --- | --- | --- | --- |
| **Antibiotics** |  |  |  |  |
| ELA01a^b^ | 49952 | 26842 | 24925 | 343 |
| ELA01b^c^ | 44454 | 26438 | 18792 | 341 |
| ELA01c^d^ | 12939 | 6705 | 6847 | 339 |
| ELA02b | 42897 | 23064 | 20507 | 343 |
| ELA02c | 11260 | 5063 | 6388 | 338 |
| ELA03a | 12738 | 5804 | 7115 | 347 |
| ELA03b | 14659 | 6567 | 8330 | 335 |
| ELA03c | 17904 | 7463 | 10618 | 341 |
| ELA33a | 10144 | 3334 | 6900 | 353 |
| ELA33b | 9937 | 7096 | 3057 | 321 |
| ELA33C | 22350 | 13397 | 10226 | 340 |
|  |  |  |  |  |
| **Controls** |  |  |  |  |
| ELA04a | 34868 | 21301 | 14864 | 334 |
| ELA04b | 43543 | 21905 | 22789 | 345 |
| ELA04c | 21880 | 11345 | 10725 | 343 |
| ELA07a | 19339 | 13998 | 6023 | 339 |
| ELA07b | 29280 | 14615 | 15437 | 342 |
| ELA07c | 23568 | 11778 | 12307 | 342 |
| ELA08a | 40289 | 23032 | 18098 | 336 |
| ELA08b | 51331 | 33732 | 18605 | 335 |
| ELA08c | 14123 | 6708 | 7745 | 348 |
| ELA09a | 13865 | 7349 | 7187 | 345 |
| ELA09b | 18759 | 13128 | 6528 | 335 |
| ELA09c | 26927 | 18302 | 9239 | 333 |
| ELA100a | 23040 | 15823 | 7951 | 337 |
| ELA100b | 17194 | 10259 | 7195 | 339 |
| ELA100c | 79938 | 55290 | 26402 | 339 |

^a^Includes trimmed reads, reads removed with length variation, and reads with homopolymers

^b^represents the Day 3 time point, ^c^represents the 2 Week time point, ^d^represents the 6 week time point

**S3B Table.** Saliva 16S rRNA reads

| Sample | Number of Reads | Trimmed or Removed^a^ | Final Read Count | Mean Length |
| --- | --- | --- | --- | --- |
| **Antibiotics** |  |  |  |  |
| ELA01a^b^ | 27222 | 17486 | 10728 | 339 |
| ELA01b^c^ | 7396 | 3337 | 4224 | 350 |
| ELA01c^d^ | 35804 | 20006 | 16595 | 337 |
| ELA02a | 26192 | 14707 | 12066 | 346 |
| ELA02b | 22392 | 22079 | 5556 | 309 |
| ELA02c | 29940 | 25939 | 6261 | 313 |
| ELA03a | 25545 | 15987 | 10038 | 337 |
| ELA03b | 40642 | 21530 | 19774 | 338 |
| ELA03c | 31184 | 19011 | 12682 | 334 |
| ELA33a | 34739 | 19862 | 17176 | 353 |
| ELA33b | 15354 | 8220 | 7396 | 346 |
| ELA33c | 13634 | 7747 | 6225 | 350 |
|  |  |  |  |  |
| **Controls** |  |  |  |  |
| ELA04a | 24806 | 15452 | 10139 | 335 |
| ELA04b | 57183 | 43780 | 15491 | 325 |
| ELA04c | 43898 | 30677 | 14684 | 331 |
| ELA07a | 14685 | 7179 | 7846 | 337 |
| ELA07b | 21658 | 13388 | 9162 | 339 |
| ELA07c | 31175 | 19315 | 12584 | 337 |
| ELA08a | 12064 | 7011 | 5531 | 330 |
| ELA08b | 17112 | 10746 | 6655 | 334 |
| ELA08c | 50461 | 35589 | 16018 | 325 |
| ELA09a | 353166 | 212188 | 153076 | 345 |
| ELA09b | 23629 | 14242 | 10030 | 344 |
| ELA09c | 22983 | 13160 | 11417 | 349 |
| ELA100b | 26375 | 17145 | 9898 | 339 |
| ELA100c | 11546 | 6993 | 4796 | 333 |

^a^Includes trimmed reads, reads removed with length variation, and reads with homopolymers

^b^represents the Day 3 time point, ^c^represents the 2 Week time point, ^d^represents the 6 week time point
